# Ultrafast spin-flip exciton conversion and narrowband sky-blue luminescence in a fused polycyclic selenaborin emitter

**DOI:** 10.3389/fchem.2024.1375552

**Published:** 2024-03-25

**Authors:** Sudhir K. Keshri, Guanting Liu, Takuma Yasuda

**Affiliations:** ^1^ Institute for Advanced Study, Kyushu University, Fukuoka, Japan; ^2^ Department of Applied Chemistry, Kyushu University, Fukuoka, Japan

**Keywords:** thermally activated delayed fluorescence, narrowband emission, selenaborin, spinorbit coupling, heavy atom effect, helicity, OLED

## Abstract

Thermally activated delayed fluorescence (TADF) materials with high photoluminescence quantum yields and fast reverse intersystem crossing (RISC) capabilities are highly desirable for applications in high-efficiency organic light-emitting diodes. Herein, we report the synthesis as well as structural and photophysical properties of 5,9-diselena-13b-boranaphtho[3,2,1-*de*]anthracene (**SeBSe**) as a narrowband-emissive TADF material. The incorporation of two selenium atoms into the boron-fused pentacyclic π-core results in a small singlet–triplet energy gap (Δ*E*_ST_) and thereby significant TADF properties. Moreover, theoretical calculations revealed a noticeable spin-orbit coupling enhancement between the singlet and triplet manifolds in **SeBSe** by virtue of the heavy-atom effect of selenium atoms. Consequently, **SeBSe** allows ultrafast spin-flip RISC with the rate constant surpassing 10^8^ s^−1^, which far exceeds the corresponding fluorescence radiative decay rate (∼10^6^ s^−1^), enabling an ideal singlet–triplet superimposed excited state.

## 1 Introduction

Thermally activated delayed fluorescence (TADF) is an emission phenomenon induced by the reverse intersystem crossing (RISC) process between the lowest excited triplet (*T*_1_) and singlet (*S*_1_) states Uoyama et al. (2012). In general, RISC involving spin-flip is the rate-limiting step in the overall TADF process; however, it can be facilitated by minimizing the energy gap (Δ*E*_ST_) and strengthening the spin-orbit coupling (SOC) between *S*_1_ and *T*_1_ in TADF systems Wada et al. (2020); Aizawa et al. (2021). In the conventionally designed TADF materials, donor (D) and acceptor (A) units are integrated in a twisting manner to spatially separate the highest occupied molecular orbital (HOMO) and lowest unoccupied molecular orbital (LUMO) onto the D and A units, respectively, hence minimizing Δ*E*_ST_ (typically below 0.2 eV) Wong and Zysman-Colman (2017); Cai and Su (2018); Liu et al. (2018). However, the intrinsic intramolecular charge transfer (ICT) characteristics inevitably cause substantial structural relaxation between the ground and excited states, resulting in broadening of the emission spectrum with a large full width at half maximum (FWHM ≥70 nm). Such broad emissions negatively affect the color purity of the emitters, particularly when considering their application in organic light-emitting diodes (OLEDs).

**FIGURE 1:**
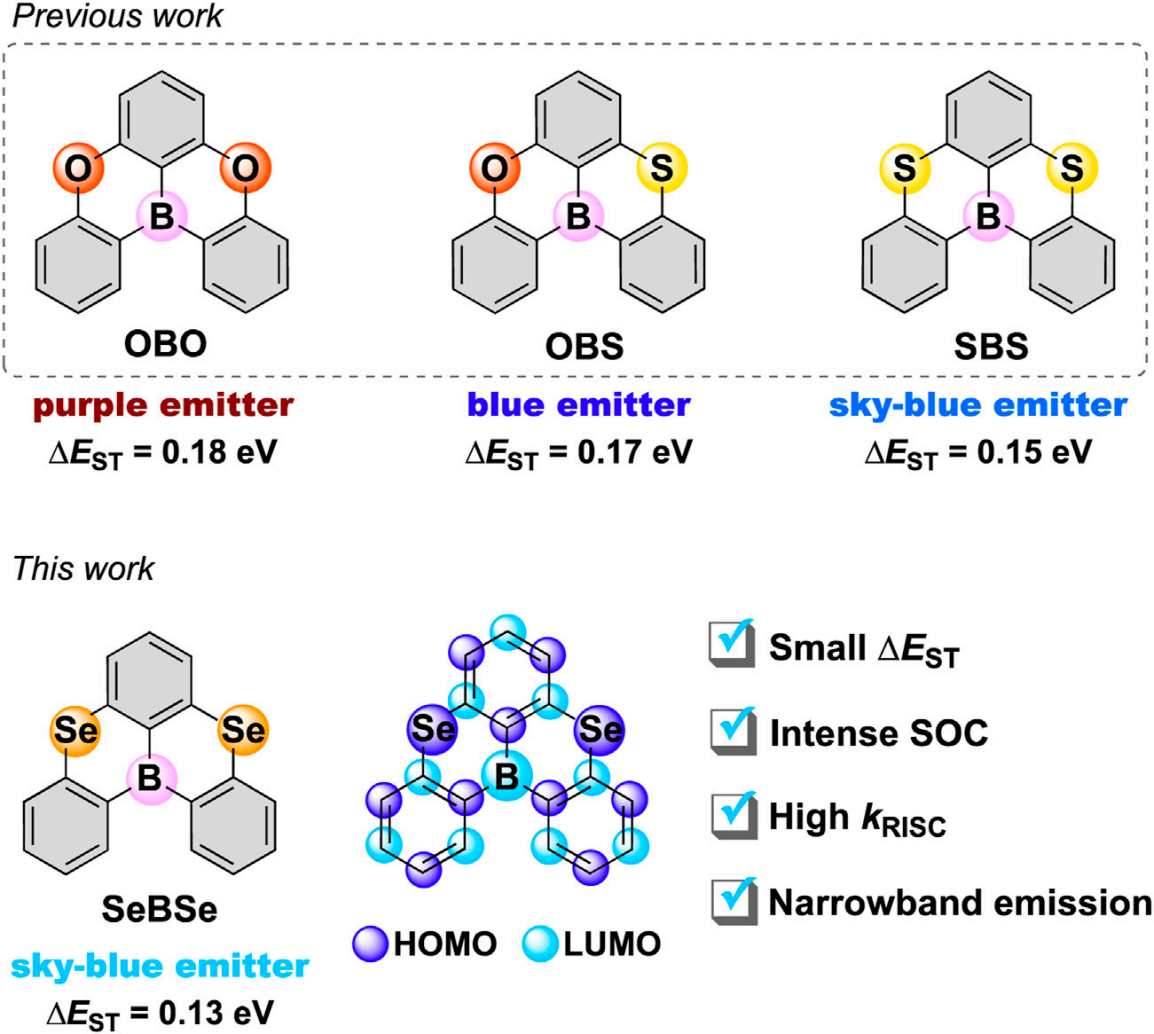
Chemical structures of the **XBX**-series MR-TADF emitters (**X** = **O**, **S**, and **Se**). The localized HOMO and LUMO in **SeBSe** are illustrated by purple and light-blue balls, respectively.

Recently, Hatakeyama et al. introduced a pioneering concept, multi-resonance (MR)-TADF, by installing electron-accepting boron (B) and electron-donating nitrogen (N) or oxygen (O) atoms at site-specific positions to induce the alternating resonance effects and attain TADF Hatakeyama et al. (2016); Hirai et al. (2015). In contrast to the conventional D–A-type TADF systems, the short-range charge transfer of MR-TADF systems based on the atomically separated HOMO and LUMO allows them to suppress structural relaxation and vibronic coupling, leading to a small Δ*E*_ST_, narrowband emissions, and high photoluminescence quantum yields Kim and Yasuda (2022). While most MR-TADF materials exhibit very slow RISC rates on the order of 10^4^ s^–1^, our group (Nagata et al., 2021; Park et al., 2022a; Park et al., 2022b) and others (Chen et al., 2021; Hu et al., 2022) have revealed that electronic perturbations from sulfur (S) or selenium (Se) atoms can significantly enhance SOC and thereby accelerate RISC in MR-TADF systems. In 2021, Chen et al. reported fused pentacyclic molecules, **OBS** and **SBS** (Figure 1), in which the O atoms of **OBO** (Hirai et al., 2015) were replaced stepwise by S atoms. They found that the incorporation of S atoms gradually decreased the Δ*E*_ST_ value and simultaneously enhanced the SOC, increasing the RISC rate up to ∼10^5^ s^−1^ Chen et al. (2021). In 2022, our group achieved a record-setting RISC rate as high as ∼10^8^ s^−1^ for a heavier Se-doped MR-TADF emitter Park et al. (2022a). As supported by recent theoretical and computational studies (Pratik et al., 2022; Hagai et al., 2024), systematic chalcogen replacement is a viable and effective approach for controlling the photophysical properties and exciton kinetics of MR-TADF systems.

Herein, we report the synthesis as well as structural and photophysical properties of 5,9-diselena-13b-boranaphtho[3,2,1-*de*]anthracene (**SeBSe**; Figure 1) as a new MR-TADF framework. The incorporation of two Se atoms into the common fused pentacyclic π-core caused a large helical distortion of the entire skeleton, resulting in structural and electronic property changes compared to the parent **OBO**. Computational simulations suggested that the SOC matrix elements of **SeBSe** were significantly enhanced compared to those of the previously reported O- and S-doped congeners (**OBO** and **SBS**). In addition, doping with heavier chalcogens led to smaller Δ*E*_ST_ values, thereby enhancing the TADF properties. **SeBSe** achieved ultrafast spin-flip RISC with a rate constant of ∼10^8^ s^−1^, which is approximately three orders of magnitude higher than that of **SBS**.

## 2 Results and discussion

### 2.1 Synthesis and structural analysis

**SCHEME 1:**
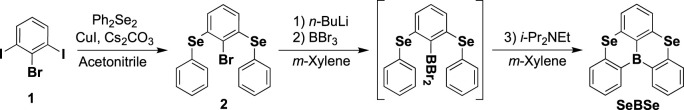
Synthetic route for **SeBSe** doped with two Se atoms.

**SeBSe** was synthesized in two steps using commercially available 2-bromo-1,3-diiodobenzene (**1**) as the starting material (Scheme 1). Precursor **2** was prepared by reacting **1** with diphenyl diselenide (Ph_2_Se_2_) in acetonitrile under reflux in the presence of a catalytic amount of copper(I) iodide and a large excess of cesium carbonate Zhang et al. (2021). The latter step is the so-called one-pot borylation (Hatakeyama et al., 2016), which consists of lithiation/substitution followed by tandem bora-Friedel-Crafts reactions. The final target, **SeBSe**, was fully characterized using ^1^H and ^13^C NMR, mass spectrometry, and single-crystal X-ray crystallography. The detailed synthesis procedures and characterization data are provided in the Supplementary Material (Supplementary Figures S1, S2).

**FIGURE 2:**
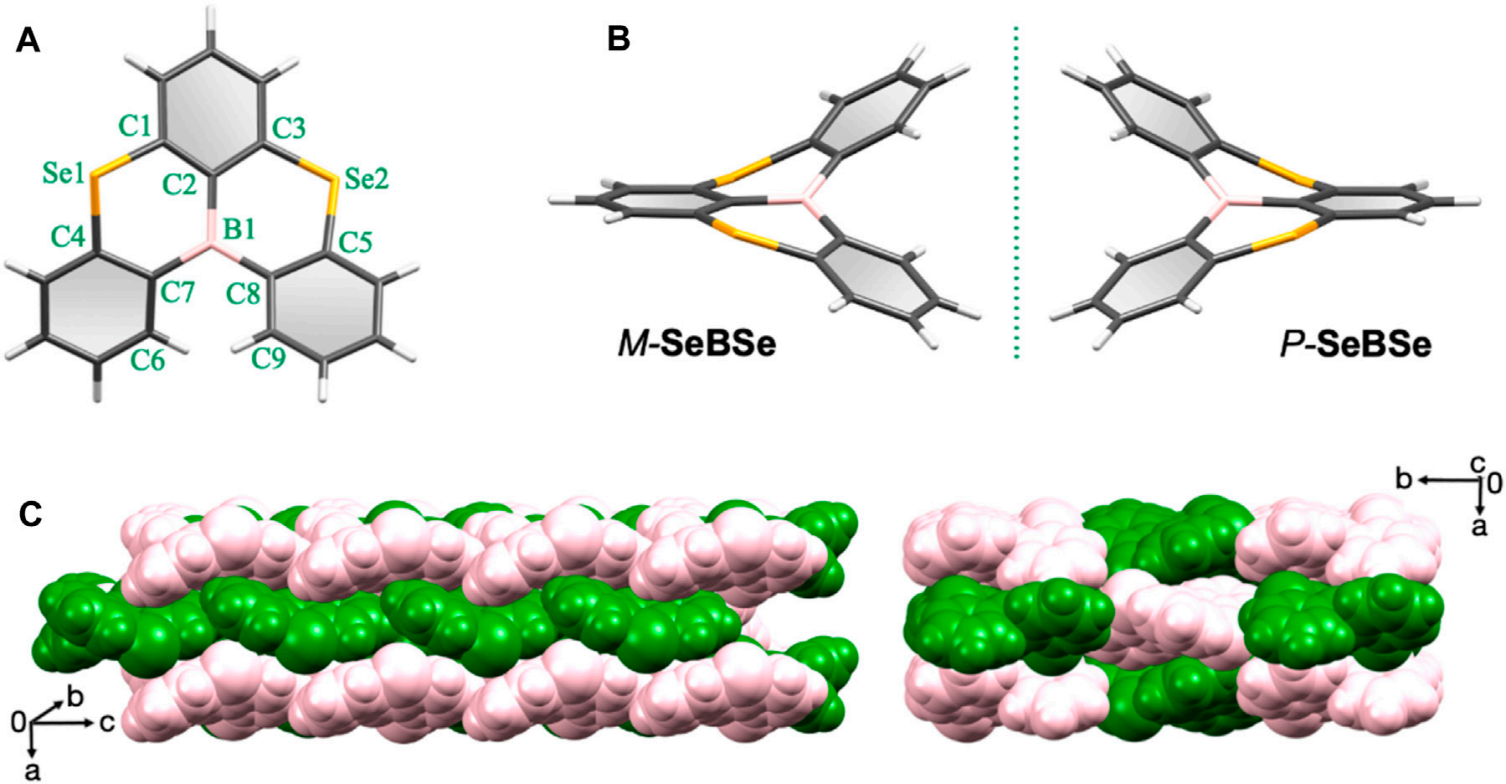
Single-crystal X-ray structures of **SeBSe** (CCDC 2326001): **(A)** top view of *M*-**SeBSe**, **(B)** side view of a helical *M*-**SeBSe** and *P*-**SeBSe** enantiopair with a mirror-image relationship, and **(C)** space-filling representations of the packing structure viewed from different angles. The *M*-**SeBSe** and *P*-**SeBSe** are drawn in green and pink colors, respectively, for clarity.

Single crystals suitable for X-ray analysis were obtained by the slow diffusion of ethanol into a chloroform solution of **SeBSe** at room temperature. Interestingly, **SeBSe** self-organized into a crystal structure with the Sohncke space group *P*2_1_2_1_2_1_, which contained no mirror nor inversion symmetry operations (Supplementary Table S1). The fact that **SeBSe** without a chiral center crystallizes in such a non-centrosymmetric space group can be related to its molecular helicity. Crystallographic analysis revealed that **SeBSe** adopts a highly distorted nonplanar structure, forming a pair of helical enantiomers (Figures 2A, B) because of the bond length mismatch caused by the rather long C–Se bonds and intramolecular steric repulsion between the adjacent benzene rings. As indicated by the dihedral angles (*φ* = ∠C6–C7–C8–C9) around the helicity, the right-handed helicene (*P*-**SeBSe**, *φ* = +53.2°) seems to be slightly more distorted compared to the left-handed one (*M*-**SeBSe**, *φ* = −52.7°). The bond angles ∠C1–Se1–C4 and ∠C3–Se2–C5 are 100.3° and 99.9° for *M*-**SeBSe**, while they are 99.6° and 103° for *P*-**SeBSe**. For *M*-**SeBSe**, all four C–Se bonds fall in the range of 1.88–1.94 Å, suggesting single bond character. In the case of the more distorted *P*-**SeBSe**, the outer C4–Se1 and C5–Se2 bonds (2.00–2.03 Å) are elongated, whereas the inner C1–Se1 and C3–Se2 bonds (1.71–1.80 Å) are contracted. Therefore, **SeBSe** inevitably prefers to form helicene structures rather than planar structures because these C–Se bonds are substantially longer than the C–B bonds (1.54–1.57 Å). As illustrated in Figure 2C, *M*-**SeBSe** (green) and *P*-**SeBSe** (pink) enantiopairs are alternately arranged and closely packed via noncovalent C–H···*π*_(C)_ and C–H···*n*_(Se)_ interactions in the crystals.

### 2.2 Computational simulations

The ground-state (*S*_0_) geometries of **SeBSe** and its congeners (**SBS** and **OBO**) were optimized using density functional theory (DFT) calculations at the B3LYP/6-31G(d) level (Supplementary Figure S3). In contrast to the fully planar **OBO**, the computed *S*_0_ state of **SeBSe** adopted a helically distorted structure, as expected, which is in good agreement with the single-crystal structure. The dihedral angle around the helicity (*φ*) was estimated to be 50.8°, which is comparable to that observed in the single-crystal structure (∼53°). To gain insight into the dynamic helicity inversion (*i.e.*, *M*-**SeBSe** ⇄ *P*-**SeBSe**), potential energy surface scans were performed for **SeBSe** by varying the degree of *φ* using the DFT method (Supplementary Figure S4). A nearly planar conformer of **SeBSe** has the highest energy, with an energy barrier of ∼69 kJ mol^−1^ for helicity inversion. Hence, we attempted optical resolution using preparative HPLC equipped with a chiral column but were unable to separate each helical enantiomer under multiple sets of conditions. Unlike in the solid states, helicity interconversion (or racemization) of **SeBSe** may occur rapidly in solution at room temperature.

**FIGURE 3:**
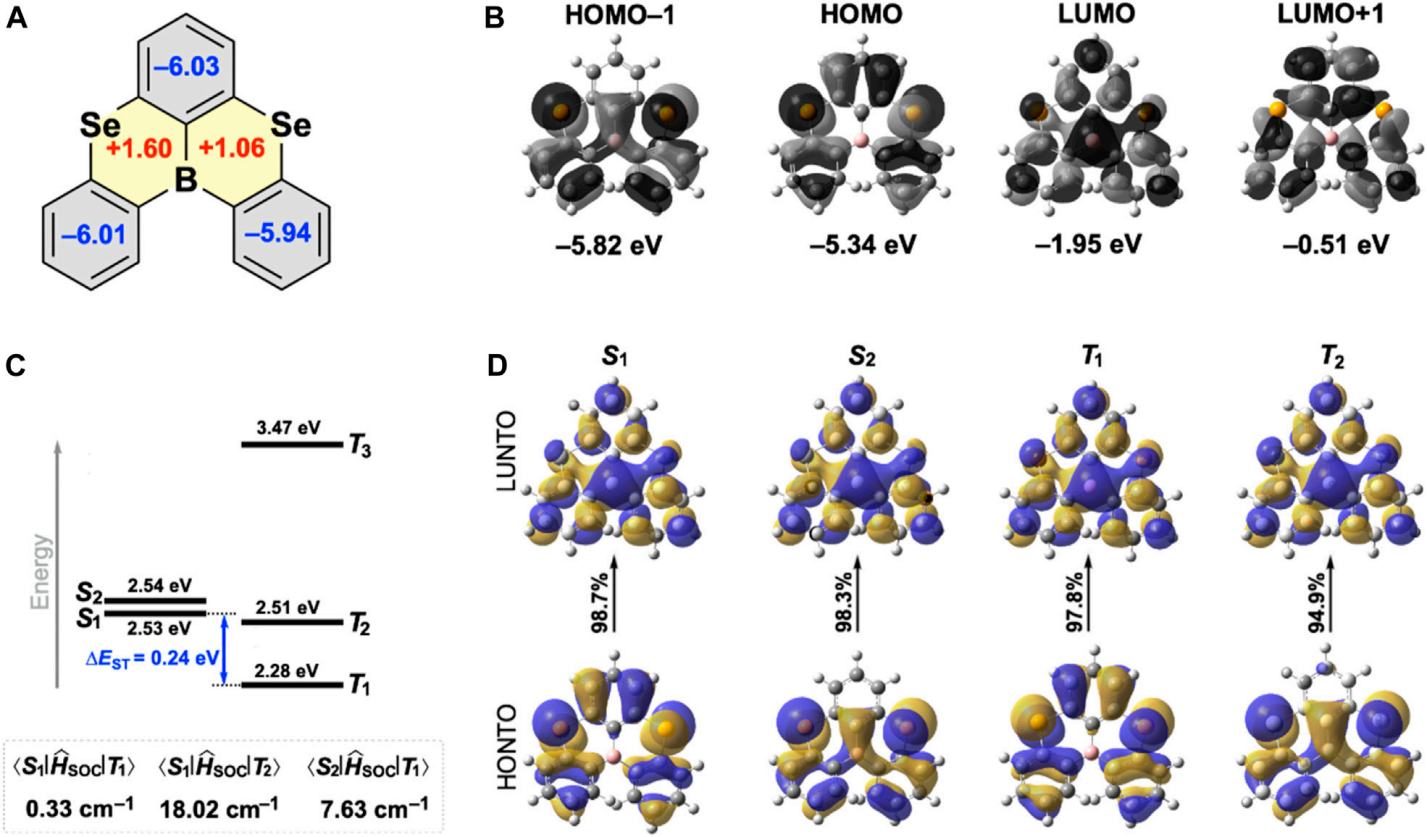
**(A)** NICS(0) values for **SeBSe** calculated at the B3LYP/6-311+G(d,p)//B3LYP/6-31G(d) level; the aromatic and non-aromatic rings are drawn in gray and yellow, respectively. **(B)** FMOs, **(C)** simulated energy-level diagram with the associated SOC matrix elements, and **(D)** NTOs for **SeBSe** calculated at the B3LYP/6-31G(d) level.

To understand the nature of each ring comprising **SeBSe**, we calculated the nucleus-independent chemical shifts (NICS) (Chen et al., 2005) at the geometrical center of the ring (NICS(0)) (Figure 3A). The large negative NICS(0) values of the three peripheral benzene rings (−5.9 to −6.0) indicate the presence of induced diatropic ring currents owing to aromaticity, whereas the two selenaborin rings exhibit small positive NICS(0) values (+1.6 and +1.1) attributable to non-aromaticity.

As with common MR-TADF molecules, the Frontier molecular orbitals (FMOs) of **SeBSe** were characterized by significant localization on the constituent atoms (Figure 3B). Despite the helically distorted structure, the HOMO and LUMO of **SeBSe** are extended throughout the molecule with complementary spatial distributions, where the Se and B atoms intensively contribute to inducing the MR effects. Although similar FMO patterns were observed in **OBO** and **SBS**, the electron density distribution on the two chalcogen atoms increased significantly when changing from O to S and Se (Figure 3B and Supplementary Figure S5). The calculated HOMO energy levels gradually increased in the order of **OBO**, **SBS**, and **SeBSe** (−5.62, −5.44, and −5.34 eV, respectively), whereas the LUMO energy levels decreased in the same order (−1.67, −1.90, and −1.95 eV, respectively). Accordingly, the HOMO–LUMO gap of **SeBSe** (3.39 eV) is considerably smaller than those of **SBS** (3.54 eV) and **OBO** (3.95 eV) and therefore, **SeBSe** is expected to emit at a longer wavelength (or lower energy).

We further computed and analyzed the energy landscape and natural transition orbitals (NTOs) of the excited singlet and triplet states. For **SeBSe**, the two lowest excited singlet states (*S*_1_ and *S*_2_) were nearly degenerate and energetically close to the higher-order triplet state (*T*_2_) (Figure 3C). This configuration is suitable for effectively harvesting the radiative *S*_1_ state through various channels from *T*_1_, *T*_2_, and *S*_2_. The NTO analysis of **SeBSe** (Figure 3D) indicates that the highest occupied NTOs (HONTOs) for *S*_1_ and *T*_1_ are very similar to the HOMO, while the HONTOs for *S*_2_ and *T*_2_ correspond to HOMO−1. The lowest unoccupied NTOs (LUNTOs) for these excited states are predominantly characterized by the LUMO. We also calculated the SOC matrix elements ( $\langle S\left|{Ĥ}_{\text{SOC}}\right|T\rangle$ ) for **SeBSe** and its congeners (Figure 3C and Supplementary Figure S6). For **SeBSe**, the calculated $\langle S_{1}\left|{Ĥ}_{\text{SOC}}\right|T_{1}\rangle$ value was not zero but relatively small (∼0.3 cm^−1^), reflecting the minimal change in orbital angular momentum between *T*_1_ and *S*_1_. However, the SOC matrix elements between *T*_1_ and *S*_2_ ( $\langle S_{2}\left|{Ĥ}_{\text{SOC}}\right|T_{1}\rangle$ ∼7.6 cm^−1^) as well as *T*_2_ and *S*_1_ ( $\langle S_{1}\left|{Ĥ}_{\text{SOC}}\right|T_{2}\rangle$ ∼18.0 cm^−1^) were significantly enhanced, presumably because of the synergistic effect of the heavy Se atoms and large orbital angular momentum changes. As a result, SOC enhancement should promote exciton spin interconversion and hence increase the RISC rate constant (*k*_RISC_), as rationalized by the following relationship: $k_{\text{RISC}}\propto {\langle \left.S\right|{Ĥ}_{\text{SOC}}\left|T\right.\rangle }^{2}/{\Delta E}_{\text{ST}}$ .

### 2.3 Photophysical properties and kinetics

**FIGURE 4:**
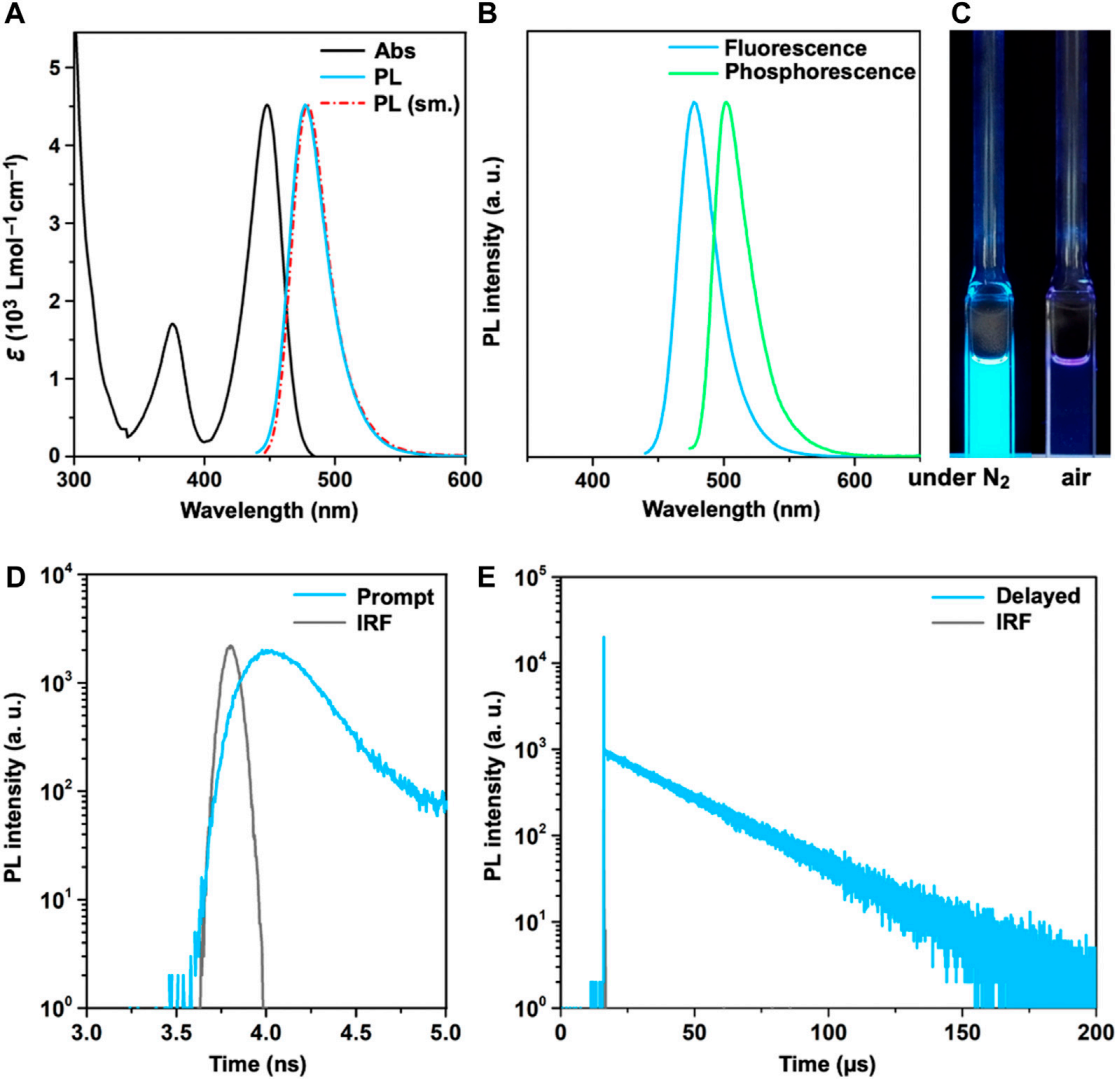
**(A)** UV/Vis absorption (black) and PL spectra (sky-blue) of **SeBSe** in a deoxygenated toluene solution (10^–5^ M) at 300 K, together with its simulated PL profile (red); **(B)** normalized fluorescence and phosphorescence spectra of the **SeBSe** solution measured at 300 and 77 K, respectively; **(C)** photograph showing sky-blue emission under UV illumination at 365 nm; transient PL decay profiles of the **SeBSe** solution in **(D)** nanosecond and **(E)** microsecond regime. The instrument response function (IRF) is shown as gray lines in **(D, E)**.

Figure 4 shows the basic photophysical properties of **SeBSe** in a dilute toluene solution, and Table 1 summarizes the relevant data. **SeBSe** exhibited an intense absorption peak (*λ*_abs_) at 448 nm, which can be attributed to the HOMO→LUMO electronic transition. This main absorption band is considerably red-shifted compared to those reported for **OBO** (*λ*_abs_ = 378 nm) and **SBS** (*λ*_abs_ = 431 nm) (Chen et al., 2021), which is in agreement with the results of their computationally simulated absorption spectra (Supplementary Figure S7). The deoxygenated solution of **SeBSe** exhibited strong sky-blue photoluminescence (PL) with an emission peak (*λ*_PL_) at 477 nm and absolute quantum yield (*Φ*_PL_) of 71%. Similar to the absorption profile, the emission band of **SeBSe** was red-shifted with respect to those of **OBO** and **SBS** (*λ*_PL_ = 396 and 457 nm, respectively) but maintained a narrow spectral FWHM of 34 nm (0.18 eV). The PL emission of **SeBSe** was completely quenched in an aerated solution (Figure 4C), suggesting that the ISC (*S*_1_→*T*_1_) and subsequent exciton quenching by triplet oxygen is much faster than the fluorescence radiative process (*S*_1_→*S*_0_). The *S*_1_ and *T*_1_ excitation energies (*E*_S_ and *E*_T_) of **SeBSe** were estimated to be 2.60 and 2.47 eV, respectively, from the fluorescence and phosphorescence peaks (Figure 4B), resulting in a Δ*E*_ST_ of 0.13 eV. This value is smaller than those of **OBO** (Δ*E*_ST_ = 0.18 eV) and **SBS** (Δ*E*_ST_ = 0.15 eV) (Chen et al., 2021), suggesting that heavier chalcogen doping reduces the Δ*E*_ST_.

**TABLE 1:** Photophysical data of SeBSe and CzBSe.

Emitter	State^[a]^	*λ*_abs_ (nm)	*λ*_PL_ (nm)	FWHM^[b]^ (nm)	*∆E*_ST_^[c]^ (eV)	*Φ*_PL_^[d]^ (%)	*τ*_p_^[e]^ (ns)	*τ*_d_^[e]^ (μs)	*k*_r_^[f]^ (10^6^ s^–1^)	*k*_ISC_^[f]^ (10^9^ s^–1^)	*k*_RISC_^[f]^ (10^8^ s^–1^)
**SeBSe**	Sol	448	477	34	0.13	71	0.2	27.7	1.1	4.7	1.2
	Film	–	481	37	0.15	73	0.2	34.9	1.7	4.7	0.6
**CzBSe** ^[g]^	Sol	451	477	33	0.12	98	0.7	17.0	0.5	1.2	1.5
	Film	–	479	34	0.15	98	0.8	14.0	0.5	1.1	1.8

[a]Sol = deoxygenated toluene solution (10^−5^ M) at 300 K; Film = 1 wt%-doped film in mCBP, host at 300 K.

[b]Full width at half maximum of the PL, spectrum.

[c]Singlet–triplet energy gap estimated from the fluorescence and phosphorescence peak positions.

[d]Absolute PL, quantum yield evaluated using an integrating sphere.

[e]Emission lifetimes of prompt (*τ*_p_) and delayed (*τ*_d_) components.

[f]Rate constants for radiative decay (*k*_r_), ISC (*k*_ISC_), and RISC (*k*_RISC_) estimated by the reported method (Park et al., 2022a).

[g]Extracted from ref. Park et al. (2022a).

The transient PL properties highlight the unique exciton kinetics of **SeBSe**, which are somewhat different from typical TADF (Figures 4D, E). The emission component in the nanosecond regime (typically attributed to prompt fluorescence) was almost negligible, as characterized by an extremely small fractional quantum yield (<0.1%) and short picosecond-order lifetime (*τ*_p_ ∼200 ps). In contrast, the emission component in the microsecond regime (usually regarded as delayed fluorescence) dominated the overall *Φ*_PL_. **SeBSe** thus demonstrated a quasi-single-component transient PL decay with a lifetime (*τ*_d_) of 27.8 μs, which is markedly different from the two-component behavior commonly observed in TADF. This implies that the spin-flip ISC/RISC cycles were drastically accelerated by Se doping, making them much faster than the competing fluorescence radiative process. Following a recent method for exciton kinetic analysis (Park et al., 2022a), we estimated the photophysical rate constants for the fluorescence radiative decay, ISC, and RISC (*k*_r_, *k*_ISC_, and *k*_RISC_, respectively; Table 1). As expected, the *k*_RISC_ of **SeBSe** reached 1.2 × 10^8^ s^−1^, which is nearly three orders of magnitude higher than that of **SBS** (Chen et al., 2021), as a consequence of enhanced SOC and reduced Δ*E*_ST_. Furthermore, the *k*_RISC_ of **SeBSe** was approximately two orders of magnitude higher than its *k*_r_ (1.1 × 10^6^ s^−1^), indicating that the rate-limiting process was no longer RISC but the fluorescence radiative process. This observation is reminiscent of metal-TADF emitters, where the *k*_ISC_ (typically 10^9^–10^11^ s^–1^) is much faster than *k*_r_ owing to the large SOC imparted by the metal ions. To et al. (2020); Li et al. (2022) The fast ICT characteristics in metal-TADF emitters allow for equilibration of the lowest energy singlet and triplet excited states. Consequently, the emission decays of metal-TADF emitters typically show only a delayed component as a single exponential signal in the microsecond range, similar to the emission observed in phosphorescent organometallic complexes.

We also measured the steady-state and transient PL characteristics of **SeBSe** in doped thin films using 3,3′-di(carbazole-9-yl)-1,1′-biphenyl (mCBP) as the host matrix (Table 1 and Supplementary Figure S8). The photophysical properties of the **SeBSe** doped films agreed with those measured as solutions, still retaining high *Φ*_PL_ and *k*_RISC_ values as well as the narrowband emission.

### 2.4 Electroluminescence performance

**FIGURE 5:**
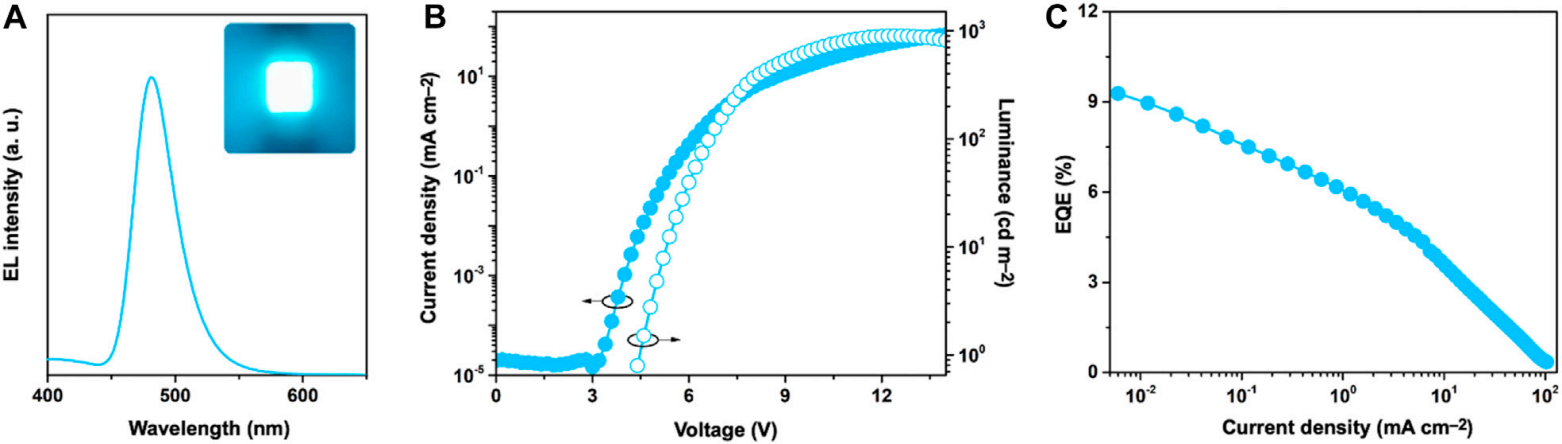
**(A)** EL spectrum (inset: EL emission color image) measured at 100 cd m^–2^, **(B)** current density–voltage–luminance (*J*–*V*–*L*) characteristics, and **(C)** external EL quantum efficiency (EQE)–current density (*J*) plot of **SeBSe**-based device.

To evaluate the electroluminescence (EL) performance of **SeBSe**, we fabricated OLEDs with the following device structure: indium tin oxide (ITO, 50 nm)/2,3,6,7,10,11-hexacyano-1,4,5,8,9,12-hexaazatriphenylene (HAT-CN, 10 nm)/1,1-bis[(di-4-tolylamino)phenyl]cyclohexane (TAPC, 40 nm)/1,3-bis(1,8-dimethylcarbazol-9-yl)benzene (mMCP, 5 nm)/1 wt%-**SeBSe** doped in 3,3′-di(carbazole-9-yl)-1,1′-biphenyl (mCBP) (30 nm)/2,8-bis(diphenylphosphinyl)dibenzo[*b,d*]furan (PPF, 5 nm)/1,3-bis[3,5-di(pyridin-3-yl)phenyl]benzene (B3PyPB, 40 nm)/8-quinolinolato lithium (Liq, 1 nm)/Al (100 nm). As depicted in Figure 5A, the **SeBSe**-based OLED exhibited narrowband sky-blue EL, with an emission peak (*λ*_EL_) at 481 nm and the CIE chromaticity coordinates of (0.106, 0.241). The EL spectrum coincided well with the corresponding PL spectrum and retained a narrowband feature. The **SeBSe**-based device exhibited a maximum external quantum efficiency (EQE_max_) of 9.3% (Figure 5C), maximum current efficiency (CE_max_) of 13.3 cd A^–1^, and maximum power efficiency (PE_max_) of 9.5 lm W^−1^ (Supplementary Figure S9).

## 3 Conclusion

In this study, we developed a novel Se-doped pentacyclic organoboron emitter (**SeBSe**) and investigated its structural and photophysical properties. The introduction of heavier Se atoms caused **SeBSe** to adopt a helically distorted structure. Using spectroscopic analysis, we also demonstrated the ultrafast spin conversion properties of **SeBSe** with a RISC rate exceeding 10^8^ s^−1^. Consequently, **SeBSe** exhibited efficient sky-blue narrowband emission consisting of a quasi-single component from the singlet–triplet superimposed excited state. Currently, pure organic emitters capable of ultrafast spin conversion and excited-state superposition are extremely rare Aizawa et al. (2021); Park et al. (2022a). The **SeBSe**-based OLED exhibited narrowband sky-blue EL with a maximum external quantum efficiency of 9.3%.

## Data Availability

The datasets presented in this study can be found in online repositories. The names of the repository/repositories and accession number(s) can be found in the article/Supplementary Material.

## References

[B1] Aizawa N. Matsumoto A. Yasuda A. (2021). Thermal equilibration between singlet and triplet excited states in organic fluorophore for submicrosecond delayed fluorescence. Sci. Adv. 7, eabe5769. 10.1126/sciadv.abe5769 33579700 PMC7880586

[B2] Cai X. Su S.-J. (2018). Marching toward highly efficient, pure-blue, and stable thermally activated delayed fluorescent organic light-emitting diodes. Adv. Funct. Mater. 28, 1802558. 10.1002/adfm.201802558

[B3] Chen F. Zhao L. Wang X. Yang Q. Li W. Tian H. (2021). Novel boron- and sulfur-doped polycyclic aromatic hydrocarbon as multiple resonance emitter for ultrapure blue thermally activated delayed fluorescence polymers. Sci. China. Chem. 64, 547–551. 10.1007/s11426-020-9944-1

[B4] Chen Z. Wannere C. S. Corminboeuf C. Puchta R. von Ragué Schleyer P. (2005). Nucleus-independent chemical shifts (NICS) as an aromaticity criterion. Chem. Rev. 105, 3842–3888. 10.1021/cr030088+ 16218569

[B5] Hagai M. Inai N. Yasuda T. Fujimoto K. J. Yanai T. (2024). Extended theoretical modeling of reverse intersystem crossing for thermally activated delayed fluorescence materials. Sci. Adv. 10, eadk3219. 10.1126/sciadv.adk3219 38295171 PMC10830100

[B6] Hatakeyama T. Shiren K. Nakajima K. Nomura S. Nakatsuka S. Kinoshita K. (2016). Ultrapure blue thermally activated delayed fluorescence molecules: efficient HOMO–LUMO separation by the multiple resonance effect. Adv. Mater. 28, 2777–2781. 10.1002/adma.201505491 26865384

[B7] Hirai H. Nakajima K. Nakatsuka S. Shiren K. Ni J. Nomura S. (2015). One-step borylation of 1,3-diaryloxybenzenes towards efficient materials for organic light-emitting diodes. Angew. Chem. Int. Ed. 54, 13581–13585. 10.1002/anie.201506335 26380959

[B8] Hu Y. X. Miao J. Hua T. Huang Z. Qi Y. Zou Y. (2022). Efficient selenium-integrated TADF OLEDs with reduced roll-off. Nat. Phot. 16, 803–810. 10.1038/s41566-022-01083-y

[B9] Kim H. J. Yasuda T. (2022). Narrowband emissive thermally activated delayed fluorescence materials. Adv. Opt. Mater. 10, 2201714. 10.1002/adom.202201714

[B10] Li T.-y. Schaab J. Djurovich P. I. Thompson M. E. (2022). Toward rational design of TADF two-coordinate coinage metal complexes: understanding the relationship between natural transition orbital overlap and photophysical properties. J. Mater. Chem. C 10, 4674–4683. 10.1039/d2tc00163b

[B11] Liu Y. Li C. Ren Z. Yan S. Bryce M. R. (2018). All-organic thermally activated delayed fluorescence materials for organic light-emitting diodes. Nat. Rev. Mater. 3, 18020. 10.1038/natrevmats.2018.20

[B12] Nagata M. Min H. Watanabe E. Fukumoto H. Mizuhata Y. Tokitoh N. (2021). Fused-nonacyclic multi-resonance delayed fluorescence emitter based on ladder-thiaborin exhibiting narrowband sky-blue emission with accelerated reverse intersystem crossing. Angew. Chem. Int. Ed. 60, 20280–20285. 10.1002/anie.202108283 34268850

[B13] Park I. S. Min H. Yasuda T. (2022a). Ultrafast triplet–singlet exciton interconversion in narrowband blue organoboron emitters doped with heavy chalcogens. Angew. Chem. Int. Ed. 61, e202205684. 10.1002/anie.202205684 35618697

[B14] Park I. S. Yang M. Shibata H. Amanokura N. Yasuda T. (2022b). Achieving ultimate narrowband and ultrapure blue organic light‐emitting diodes based on polycyclo‐heteraborin multi‐resonance delayed‐fluorescence emitters. Adv. Mater. 34, 2107951. 10.1002/adma.202107951 34877725

[B15] Pratik S. M. Coropceanu V. Brédas J.-L. (2022). Purely organic emitters for multiresonant thermally activated delay fluorescence: design of highly efficient sulfur and selenium derivatives. ACS Mater. Lett. 4, 440–447. 10.1021/acsmaterialslett.1c00809

[B16] To W.-P. Cheng G. Tong G. S. M. Zhou D. Che C.-M. (2020). Recent advances in metal-TADF emitters and their application in organic light-emitting diodes. Front. Chem. 8, 653. 10.3389/fchem.2020.00653 32850666 PMC7411996

[B17] Uoyama H. Goushi K. Shizu K. Nomura H. Adachi C. (2012). Highly efficient organic light-emitting diodes from delayed fluorescence. Nature 492, 234–238. 10.1038/nature11687 23235877

[B18] Wada Y. Nakagawa H. Matsumoto S. Wakisaka Y. Kaji H. (2020). Organic light emitters exhibiting very fast reverse intersystem crossing. Nat. Phot. 14, 643–649. 10.1038/s41566-020-0667-0

[B19] Wong M. Y. Zysman-Colman E. (2017). Purely organic thermally activated delayed fluorescence materials for organic light-emitting diodes. Adv. Mater. 29, 1605444. 10.1002/adma.201605444 28256751

[B20] Zhang J. Liu K. Li J. Xie Y. Li Y. Wang X. (2021). Harnessing Se=N to develop novel fluorescent probes for visualizing the variation of endogenous hypobromous acid (HOBr) during the administration of an immunotherapeutic agent. Chem. Commun. 57, 12679–12682. 10.1039/D1CC04832E 34779461

