# Supplementary material for: Ultrafast spin-flip exciton conversion and narrowband sky-blue luminescence in a fused polycyclic selenaborin emitter
Source: Front Chem. 2024 Mar 25;12:1375552. doi: 10.3389/fchem.2024.1375552 (PMC10999597; doi:10.3389/fchem.2024.1375552)
Supplement: Supplementary file 1 [file DataSheet1.PDF]

Supplementary Material

Ultrafast spin-flip exciton conversion and narrowband sky-blue luminescence in a fused polycyclic selenaborin emitter

Sudhir K. Keshri[1*], Guanting Liu[1], and Takuma Yasuda[1,2*]

*** Correspondence:**
Sudhir K. Keshri: keshri@ifrc.kyushu-u.ac.jp
Takuma Yasuda: yasuda@ifrc.kyushu-u.ac.jp

1. Materials and general methods

All regents and solvents were purchased from Sigma-Aldrich, Tokyo Chemical Industry (TCI), or Fujifilm Wako Pure Chemical Corp., and were used without further purification unless otherwise noted. 1,3-bis(1,8-dimethylcarbazol-9-yl)benzene (mMCP),[1] and 1,3-bis(3,5-dipyrid-3-ylphenyl)benzene (B3PyPB)[2] were prepared according to the literature procedures. 2,8-Bis(diphenylphosphinyl)dibenzo[*b*,*d*]furan (PPF) was purchased from TCI and purified by temperature-gradient vacuum sublimation before use. Other OLED materials, which are 2,3,6,7,10,11-hexacyano-1,4,5,8,9,12-hexaazatriphenylene (HAT-CN, LG Chem Ltd.), 4,4'-cyclohexylidenebis[*N*,*N*-bis(4-methylphenyl)benzenamine] (TAPC, Luminescence Technology Corp.), 3,3'-di(9*H*-carbazol-9-yl)-1,1'-biphenyl (mCBP, TCI), and 8-quinolinolato lithium (Liq, e-Ray Optoelectronics Technology Co., Ltd.), were purchased and used without further purification.

NMR spectra were recorded on an Avance III 400 spectrometer (Bruker). ^{1}H and ^{13}C NMR chemical shifts were determined relative to the signals of tetramethylsilane (δ = 0.00). Matrix-assisted laser desorption ionization time-of-flight (MALDI-TOF) mass spectra were collected on an Autoflex III spectrometer (Bruker Daltonics) using dithranol as a matrix. Elemental analyses were carried out using an MT-5 analyzer (Yanaco). The final products were purified by temperature-gradient vacuum sublimation using a P-100 system (ALS) before the measurements and device fabrication.

2. Photophysical measurements

Organic thin films for photophysical measurements were deposited under high vacuum (<7 × 10^{-5} Pa) onto a quartz substrate. UV/Vis absorption and PL spectra were measured with a V-670 spectrometer (Jasco) and a FP-8600 spectrophotometer (Jasco), respectively. The absolute PL quantum yields were determined using a C13534 Quantaurus-QY Plus system (Hamamatsu Photonics). The transient PL decay measurements for the doped thin films were performed using a C11367 Quantaurus-tau fluorescence lifetime spectrometer (Hamamatsu Photonics; λ = 340 nm, pulse width = 100 ps, and repetition rate = 5 kHz) under N_2 and vacuum (<4 × 10^{-1} Pa).

3. OLED fabrication and evaluation

ITO-coated glass substrates were cleaned with acetone and isopropanol. The substrates were then subjected to UV–ozone treatment for 15 min before being loaded into an E-200 vacuum evaporation system (ALS Technology). The organic layers and a cathode Al layer were thermally evaporated on the substrates under vacuum ($<6 \times 10^{-5}$ Pa) with a deposition rate of <0.3 nm s^{-1} through a shadow mask, defining a pixel size of 0.04 cm^2. The thickness and deposition rate were monitored in situ during deposition by an oscillating quartz thickness monitor. The *J*–*V*–*L* characteristics and angle-resolved EL intensities of the fabricated OLEDs were measured using a Keithley 2400 source meter and a CS-2000 spectroradiometer (Konica Minolta).

4. Computational methods

Quantum chemical calculations based on the time-dependent density functional theory (TDDFT) were performed using the Gaussian 16 program.[3] NICS(0) values were calculated at the B3LYP/6-311+G(*d*, *p*) on the pre-optimized structure at B3LYP/6–31G(*d*) level. FMOs and NTOs were calculated at the B3LYP/6–31G(*d*) level. The energy landscape, S_1–T_1 energy gap, and SOC matrix elements were calculated by TDDFT at the B3LYP/DZP level using the ADF software.[4]

5. X-Ray crystallographic analysis

Single crystals suitable for X-ray analysis were obtained by the slow diffusion of ethanol into a chloroform solution of **SeBSe** at room temperature. Single-crystal X-ray diffraction measurements were performed on a XtaLAB Synergy-R/DW diffractometer (Rigaku) with a HyPix detector. The diffraction data were collected using Cu-Kα radiation (λ = 1.54184 Å) monochromated with CrysAlisPro 1.171.41.96a (Rigaku OD, 2021). Using OLEX2,[5] the structures were solved with the SHELXT structure solution program[6] using Intrinsic Phasing and refined with the SHELXL refinement package.[7] The crystallographic data have been deposited in the Cambridge Crystallographic Data Centre (CCDC) as supplementary publication materials (CCDC number: 2326001). These data can be obtained free of charge from the CCDC *via* www.ccdc.cam.ac.uk/data_request/cif

6. Synthesis of SeBSe

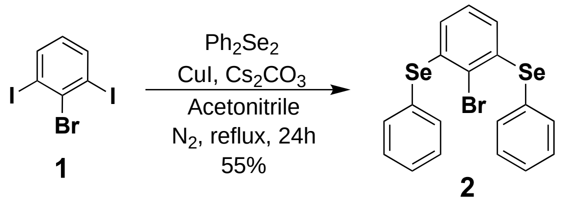

To a solution of 2-bromo-1,3-diiodobenzene (**1**) (5.00 g, 12.3 mmol), diphenyl diselenide (5.35 g, 17.2 mmol), and Cs_2CO_3 (24.00 g, 73.7 mmol) in 100 mL of anhydrous acetonitrile, copper(I) iodide (235 mg, 1.23 mmol) was added under N_2 atmosphere. The solution was refluxed for 24 h. After cooling to room temperature, the solvent was removed under reduced pressure. The crude product was purified by silica gel chromatography using hexane/dichloromethane (7/3, v/v) as eluent to give **2** as a white crystalline solid (yield = 3.13 g, 55 %). ^{1}H NMR (400 MHz, $CDCl_3$): δ 7.65 (m, 4H), 7.40 (m, 6H), 6.81 (dd, J = 7.6, 0.4 Hz, 1H), and 6.59 ppm (d, J = 8.0 Hz, 2H). ^{13}C NMR (100 MHz, $CDCl_3$): δ 138.16, 136.43, 129.87, 129.09, 129.01, 128.13, and 127.59 ppm. MS (MALDI-TOF): m/z calcd 467.15; found: 467.06 $[M]^+$.

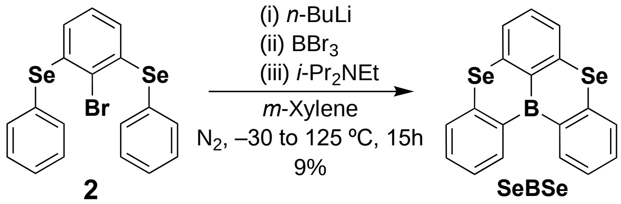

To a solution of **2** (3.12 g, 6.68 mmol) in 60 mL of anhydrous *m*-xylene, a solution of *n*-butyllithium in hexane (4.6 mL, 1.6 M, 7.35 mmol) was added dropwise at –30 °C under N_2 atmosphere. The solution was stirred at –30 °C for 1 h, and then slowly warmed to 60 ºC over 4 h. Boron tribromide (1.0 mL, 10.4 mmol) was subsequently added at 0 ºC. After aging for 15-20 min, the yellow color suspension was stirred at room temperature for 2 h. Then, *N*,*N*-diisopropylethylamine (*i*-Pr_2NEt) (2.7 mL, 15.5 mmol) was added at 0 ºC and the solution was stirred at room temperature for 20 min. Finally, the reaction mixture was stirred at 125 ºC for overnight. After cooling to room temperature, methanol was added to quench the reaction. The solvent was removed under reduced pressure to get yellow solid. The crude product was purified by silica gel chromatography using hexane/dichloromethane (10/1, v/v) as eluent and recrystallized from chloroform/methanol to give **SeBSe** as a light-yellow crystalline solid (yield = 240 mg, 9%). ^{1}H NMR (400 MHz, $CDCl_3$): δ 8.05 (dd, J = 7.6, 1.2 Hz, 2H), 7.81 (dd, J = 8.0, 0.8 Hz, 2H), 7.71 (d, J = 8.0 Hz, 2H), 7.48 (td, J = 7.6, 1.6 Hz, 2H), 7.39 (td, J = 7.6, 1.2 Hz, 2H), and 7.32 ppm (t, J = 7.6 Hz, 1H). ^{13}C NMR (100 MHz, $CDCl_3$): δ 142.70, 142.16, 140.45, 130.78, 129.73, 127.62, 125.45, and 125.00 ppm. MS (MALDI-TOF): m/z calcd 396.04; found: 396.08 $[M]^+$.

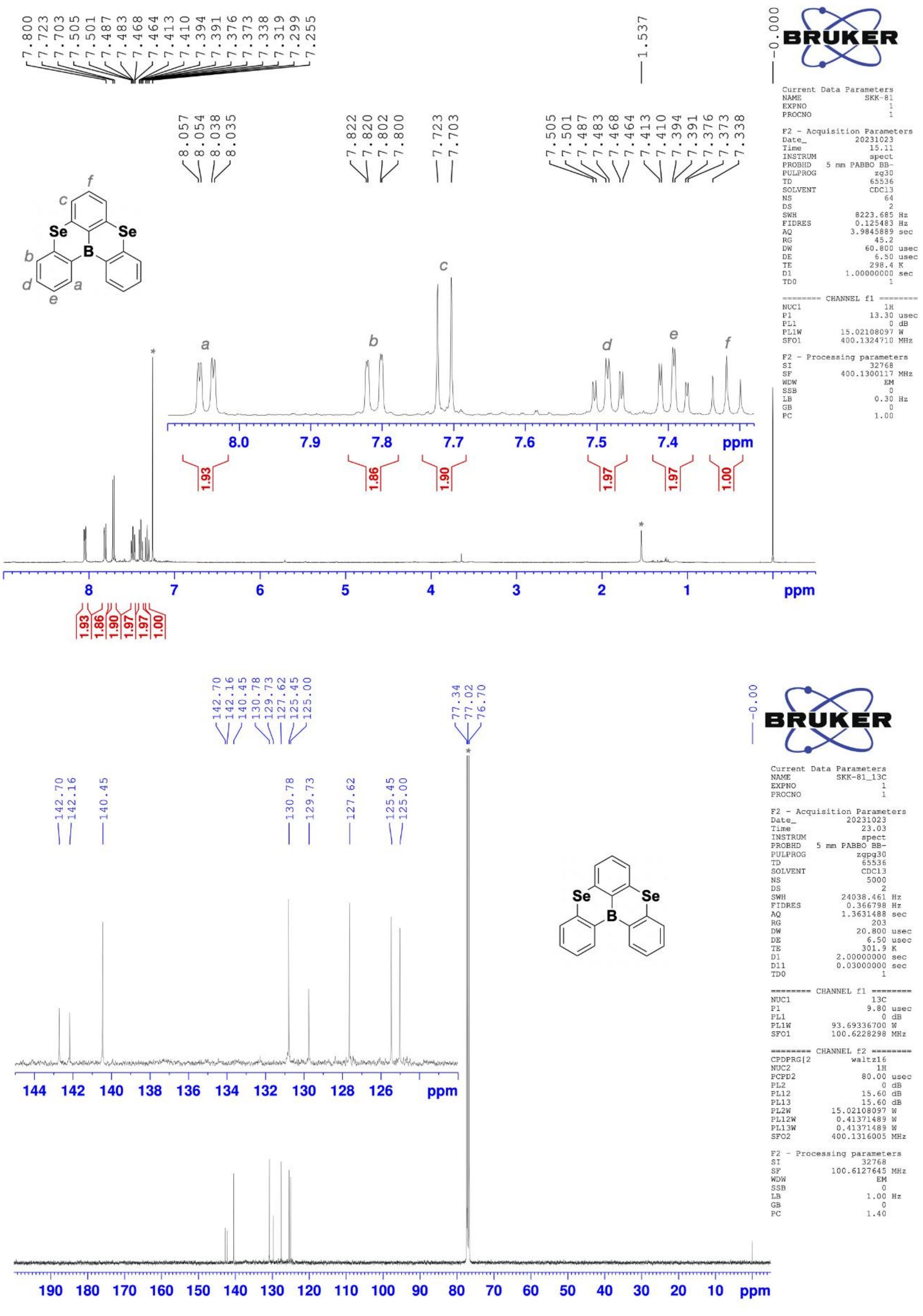

Supplementary Figure S1 ^{1}H and ^{13}C NMR spectra of **SeBSe** in $CDCl_3$ at room temperature.

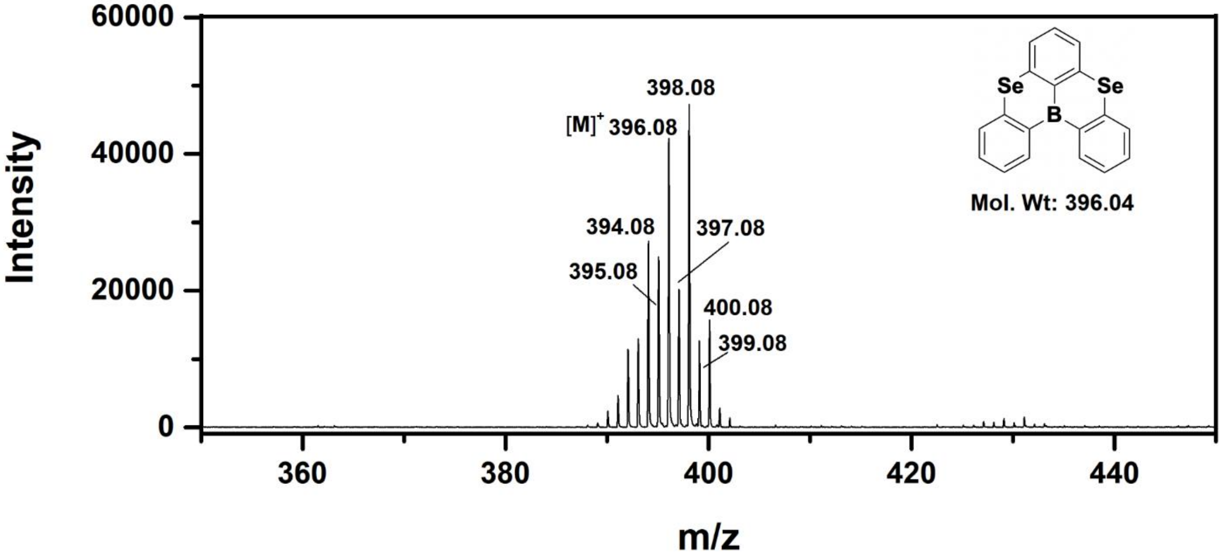

Supplementary Figure S2 MALDI-TOF MS spectrum of **SeBSe**.

Supplementary Table S1 X-ray crystallographic data for **SeBSe**

Empirical formula	$C_{18}H_{11}BSe_2$
Formula weight	396.00
Crystal system	orthorhombic
Space group	$P2_12_12_1$
Temperature (K)	100
Wavelength (Å)	1.54184
a (Å)	7.7299(2)
b (Å)	18.8834(4)
c (Å)	19.6079(4)
α (º)	90
β (º)	90
γ (º)	90
Volume (Å3)	2862.10(11)
Z	8
no. of reflections measured	18796
no. of observations	5888
no. of parameters refined	419
R1	0.0437
wR2	0.1185
GOF	1.029
CCDC no.	2326001

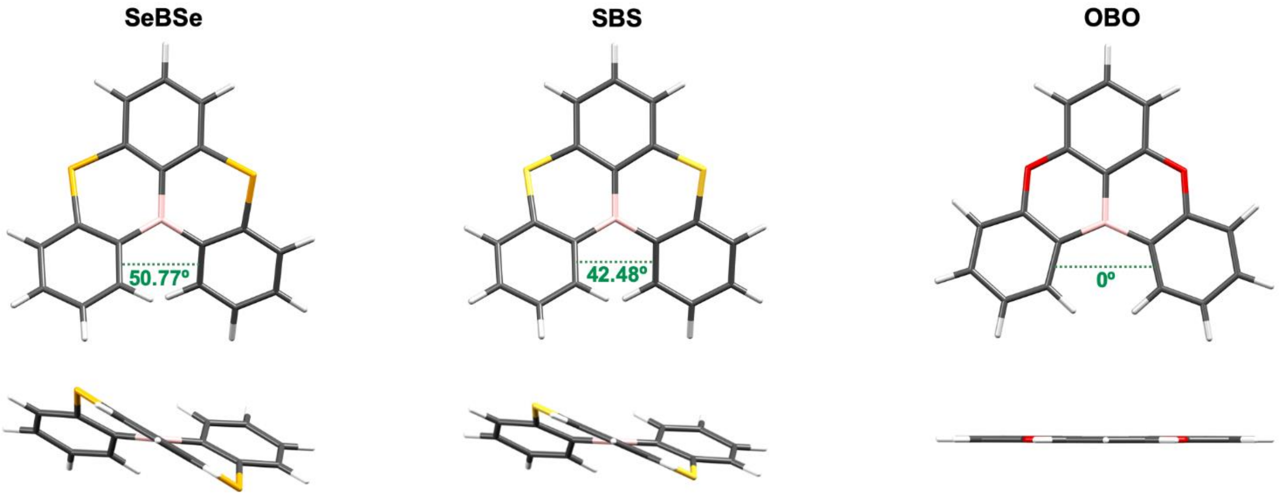

Supplementary Figure S3 Optimized ground-state geometries (top and side views) of **SeBSe**, **SBS**, and **OBO** calculated at the B3LYP/6–31G(*d*) level. The relevant dihedral angles around the helicity are also given.

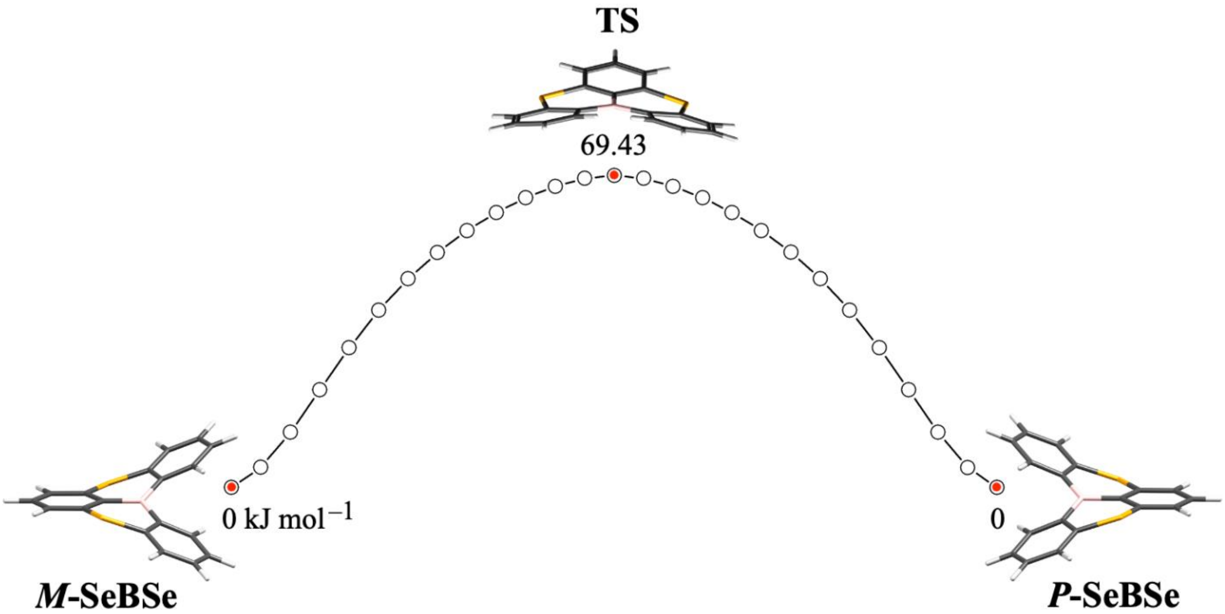

Supplementary Figure S4 Potential energy surface (PES) scan profile for plausible helicity inversion pathway in **SeBSe** calculated at the B3LYP/6–31G(*d*) level. The transition state (TS) is confirmed by the appearance of an imaginary frequency in the frequency calculation at the same level. The TS structure is nearly planar with the dihedral angle around the helicity of 0º.

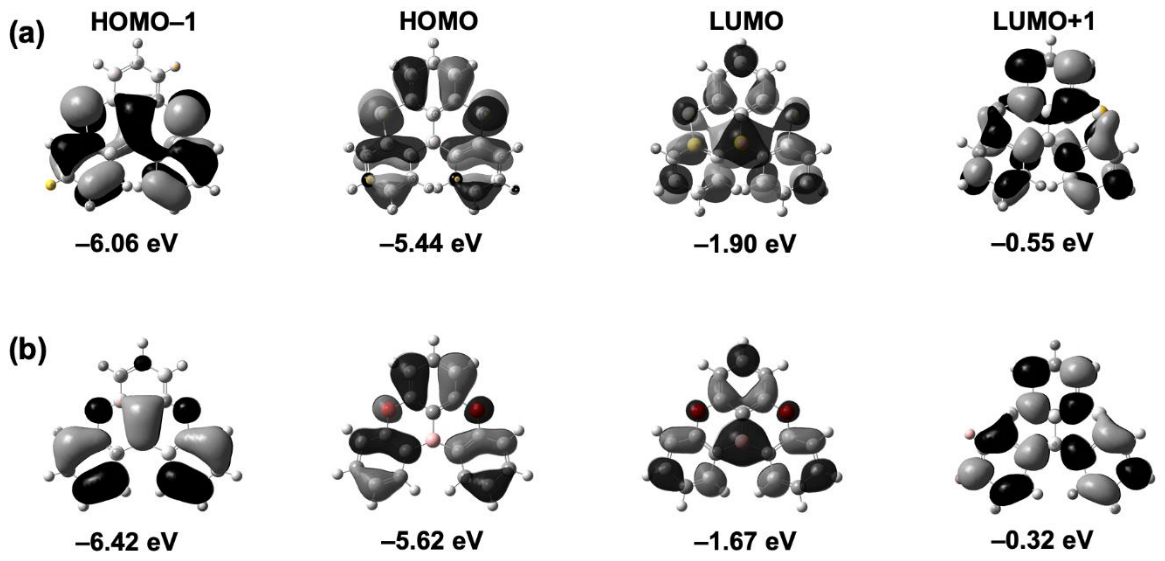

Supplementary Figure S5 FMOs of (a) **SBS** and (b) **OBO** calculated at the B3LYP/6–31G(d) level.

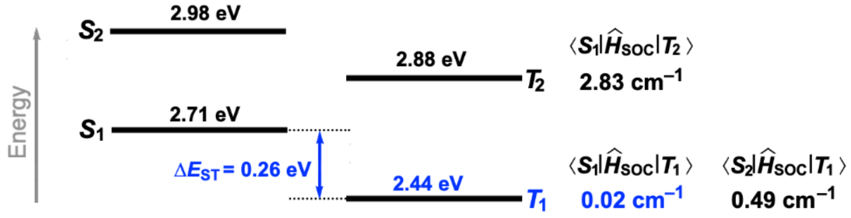

Supplementary Figure S6 Simulated energy-level diagrams of **SBS**; the SOC matrix elements are also shown.

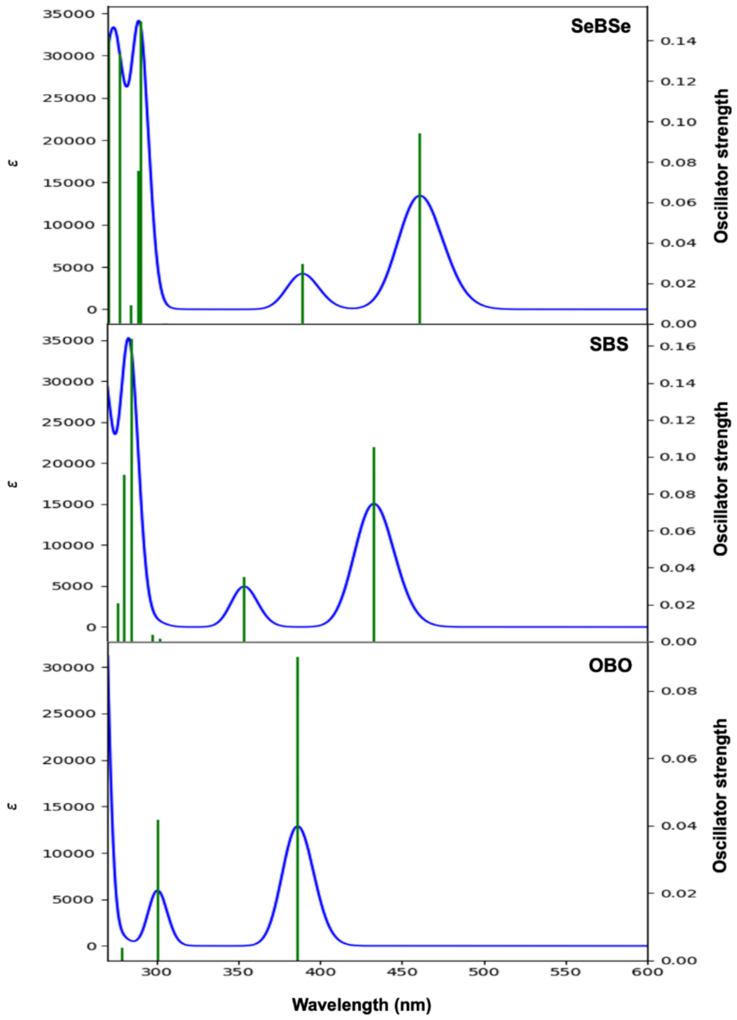

Supplementary Figure S7 Computational UV/Vis absorption spectra of **SeBSe**, **SBS**, and **OBO** calculated at B3LYP/6–31G(*d*) level.

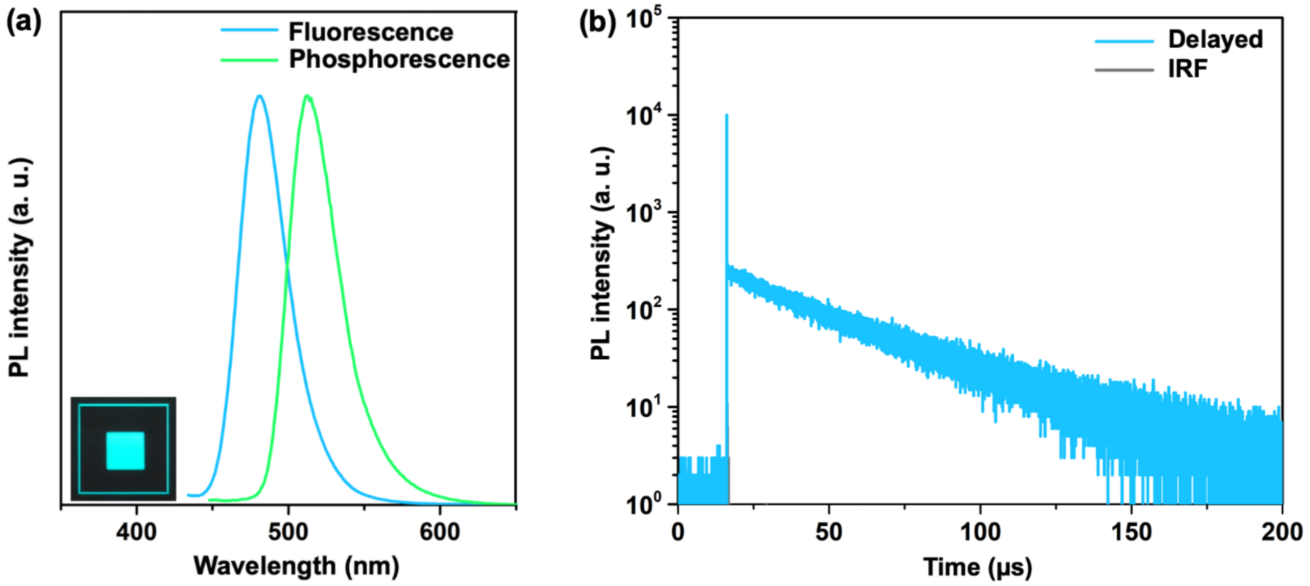

Supplementary Figure S8 (a) Normalized fluorescence and phosphorescence spectra and of a 1wt%-**SeBSe**:mCBP doped film measured at 300 and 77 K, respectively. The photograph showing sky-blue fluorescence under UV illumination at 365 nm. (b) Transient PL decay profile of a 1wt%-**SeBSe**:mCBP doped film.

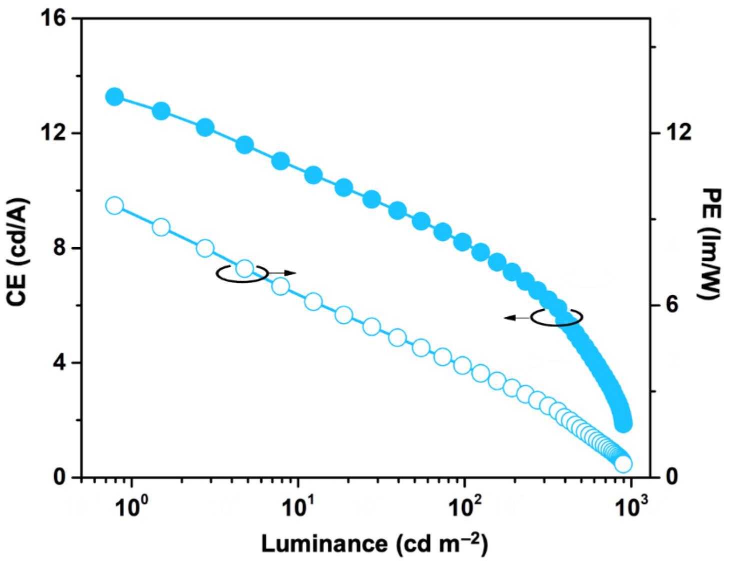

Supplementary Figure S9 Current efficiency (CE) and power efficiency (PE) versus luminance curves of an OLED with a 1wt%-**SeBSe**:mCBP emitting layer.

References

[1] Park, I. S., Min, H., Kim, J. U., Yasuda, T. *Adv. Optical Mater.* **2021**, *9*, 2101282.

[2] Sasabe, H., Gonmori, E., Chiba, T., Li, Y.-J., Tanaka, D., Su, S.-J., Takeda, T., Pu, Y.-J., Nakayama, K.-I., Kido, J. *Chem. Mater.* **2008**, *20*, 5951–5953.

[3] Frisch, M. J. et al. Gaussian, Inc., Wallingford CT, 2016.

[4] ADF2018, SCM, Theoretical Chemistry, Vrije Universiteit, Amsterdam, The Netherlands, http://www.scm.com/.

[5] Dolomanov, O. V., Bourhis, L. J., Gildea, R. J., Howard, J. A. K., Puschmann, H. *J. Appl. Cryst.* **2009**, *42*, 339–341.

[6] Sheldrick, G. M. *Acta Cryst. A* **2015**, *71*, 3–8.

[7] Sheldrick, G. M. *Acta Cryst. C* **2015**, *71*, 3–8.
